# Challenging Resection of Bilateral Parasagittal and Falcine Meningioma Involving Both Anterior Third and Middle Third of the Superior Sagittal Sinus: A Case Report and Literature Review

**DOI:** 10.7759/cureus.64865

**Published:** 2024-07-18

**Authors:** Rawia A Alzughaibi, Ghaidaa A Almuhammadi, Saud S Alasmari, Maamoun M Khoja, Aysam A Almashni

**Affiliations:** 1 College of Medicine, Taibah University, Madina, SAU; 2 College of Medicine, King Khalid University, Abha, SAU; 3 Department of Histopathology, King Salman Bin Abdulaziz Medical City, Madina, SAU; 4 Department of Neurological Surgery, King Salman Bin Abdulaziz Medical City, Madina, SAU

**Keywords:** central nervous system neoplasms, superior sagittal sinus, falx meningioma, parasagittal meningioma, meningioma

## Abstract

Meningiomas typically manifest as benign, slow-growing, and well-defined tumors on a macroscopic level and are usually asymptomatic. However, the mass effect caused by large meningiomas may lead to various neurological symptoms, commonly headaches and visual problems. Radiological imaging can establish the diagnosis, and a biopsy can provide a definitive diagnosis. Our case report describes the surgical intervention for bilateral parasagittal-falcine meningioma in a 57-year-old male who presented to the emergency department with a tonic-clonic seizure. On examination, he had a bifrontal longitudinal mass. Magnetic resonance imaging (MRI) revealed a large anterior superior falcine extra-axial mass, measuring about 5.7 x 5.3 x 3.1 cm, with surrounding vasogenic edema and superior sagittal sinus invasion. He underwent surgery for tumor resection involving the anterior third and middle third of the superior sagittal sinus without radiotherapy. He did not develop any intraoperative complications, and during the post-operative evaluation, he was symptom-free. A follow-up MRI with contrast performed three months later showed no neurological complications or recurrent tumor. To achieve better outcomes, surgical intervention for parasagittal and falcine meningiomas involving the superior sagittal sinus should aim to eliminate clinical signs, control tumor growth, and prevent neurological deterioration post-operatively.

## Introduction

Meningiomas typically manifest as benign, slow-growing, and well-defined tumors on a macroscopic level. Among intracranial meningiomas, parasagittal and falcine variants represent the second most prevalent group and are frequently encountered in neurosurgical practice [1]. Their involvement with bridging veins and major dural sinuses presents a difficult challenge during surgical intervention due to potential complications such as venous congestion, brain swelling, and venous infarction [2]. In meningiomas infiltrating both the superior sagittal sinus (SSS) and the falx, surgeons should prioritize safeguarding the SSS, bilateral cortical veins, and parafalcine veins [3].

A significant possibility is damage to the cerebral venous system during attempts to perform radical resection. This risk contributes to many cases where the tumor is not entirely removed. Consequently, some studies have described this group of meningiomas as having the highest rate of tumor re-growth. Conversely, performing radical resection of the tumor carries a higher likelihood of causing injury to the cerebral venous system and resulting in severe neurological deficits [1].

## Case presentation

A 57-year-old male was presented to the emergency department by his son, who witnessed his father having a generalized tonic-clonic seizure with up-rolling of the eyes and tongue biting for ten minutes without urine or fecal incontinence. After regaining consciousness, the patient denied any headache or muscle pain following the ictal episode. He also denied any sleep disturbances, mood changes, irritability, or headache prior to the seizure. There was no weight loss, fever, cough, headache, numbness, or weakness.

**Figure 1:**
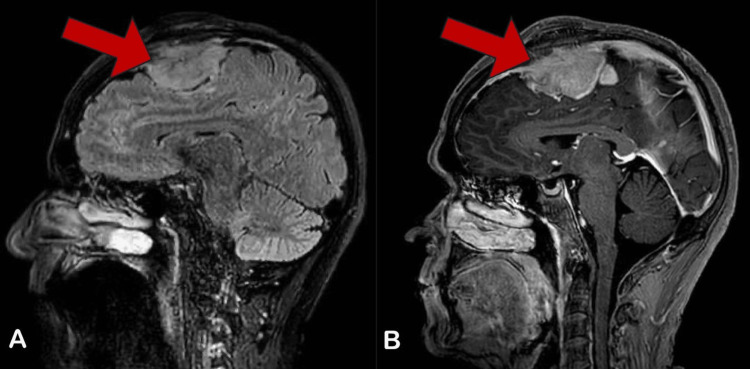
(A) Sagittal T1-Weighted MRI and (B) Sagittal T2-Weighted MRI Demonstrate a Large Anterior Superior Extra-axial Mass With a Lobulated Margin. Mass Effect on Corpus Callosum and Anterior Bodies of Lateral Ventricles Was Noted.

**Figure 2:**
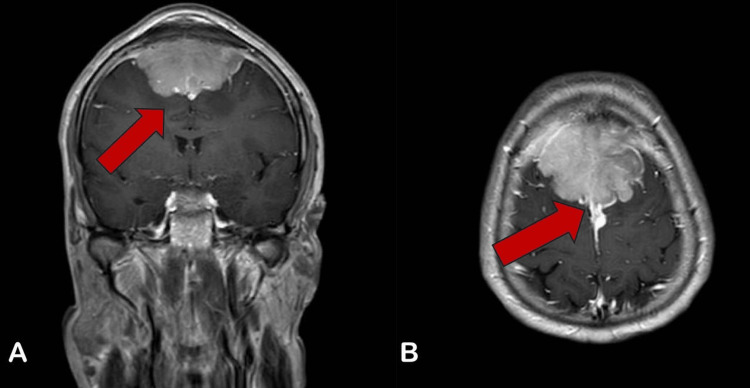
(A) Coronal T2-Weighted MRI Demonstrates the Mass Effect on the Anteromedial Portion of Bilateral Frontal Parenchyma. (B) Axial T2-Weighted MRI Shows Complete Invasion to the Middle Third of the Superior Sagittal Sinus.

On physical examination, the patient's vital signs were within normal limits; his Glasgow Coma Scale (GCS) was 15/15, pupils were equal and reactive bilaterally, and his motor power was 5/5 in his upper and lower limbs. There was a non-tender bifrontal mass with no color change, which had grown slowly over two years since he first noticed it. His initial brain computed tomography (CT) scan showed an anterior extra-axial frontal lesion with calcification and a significant mass effect. Gadolinium-enhanced magnetic resonance imaging (MRI) of the brain revealed a large 5.7 x 5.3 x 3.1 cm anterior superior falcine extra-axial mass with a lobulated margin. The mass was isointense on T1 and hyperintense on T2-weighted images, with minimal vasogenic edema noted (Figures 1, 2).

**Figure 3:**
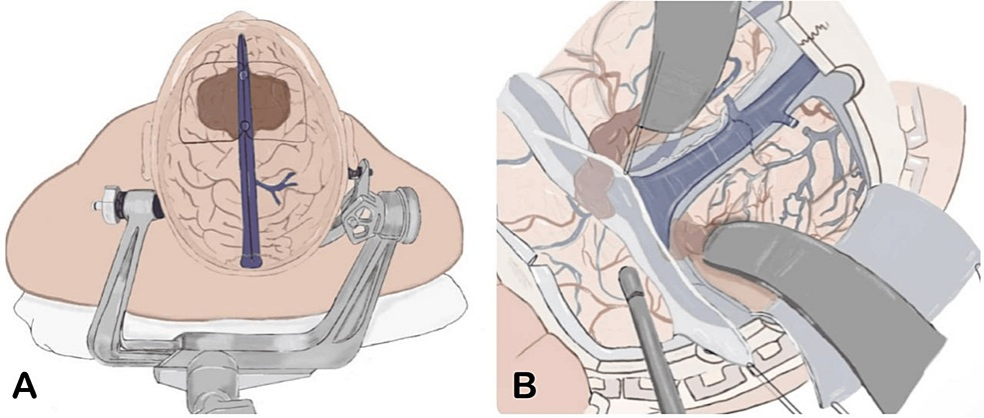
(A) Illustration of the Patient’s Head Explaining How He Was Positioned. The Midline Incision Exposes the Brain With the Superior Sagittal Sinus and Major Cortical Veins Highlighted. (B) Detailed Depiction of the Intraoperative View During the Procedure. Surgical Instruments Are Shown Retracting Brain Tissue to Provide Access to Deeper Structures, With an Emphasis on the Exposure of the Venous Sinuses and Cortical Vessels, Crucial for the Resection of the Tumor. Image Credits: Ghaidaa A. Almuhammadi

The patient underwent surgery on the third day following his initial presentation. He was placed in a supine position with the head slightly flexed, and a linear skin incision was made bi-frontally, exposing the SSS with ligation to the anterior and posterior parts of it. Complete resection of the tumor was performed from its anterior edge to its posterior edge, including the anterior third and middle third of the SSS, without leaving any residual tumor. All posterior collateral veins and supra-falcine veins were preserved. Cranioplasty was performed, replacing the infiltrated bone with a titanium mesh (Figure 3).

**Figure 4:**
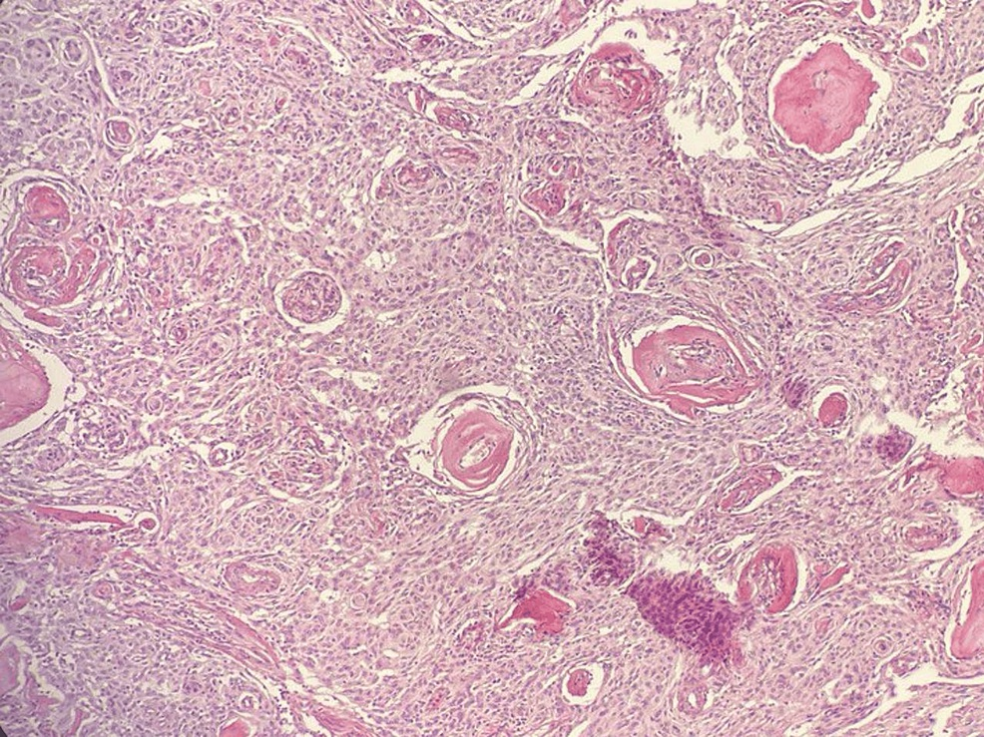
The Tumor Cells Exhibit Round Uniform Nuclei, Intranuclear Pseudo-Inclusions and Frequent Psammoma Bodies (Hematoxylin and Eosin, x100).

Histopathological microscopic examination of a tissue specimen collected during surgery showed neoplastic cell proliferation arranged in a lobulated architecture, composed of meningothelial whorls, syncytial cells with indistinct cell membranes, and eosinophilic cytoplasm. The tumor cells exhibited round uniform nuclei, intranuclear pseudo-inclusions, and frequent psammoma bodies. Foci of bone invasion were noted. Glial tissue was identified with no evidence of glial tissue invasion: no prominent nucleoli or small cells with a high nuclear-to-cytoplasmic ratio. According to the World Health Organization (WHO), the tumor was classified as grade 1. All these findings are consistent with a diagnosis of transitional meningioma (Figure 4).

**Figure 5:**
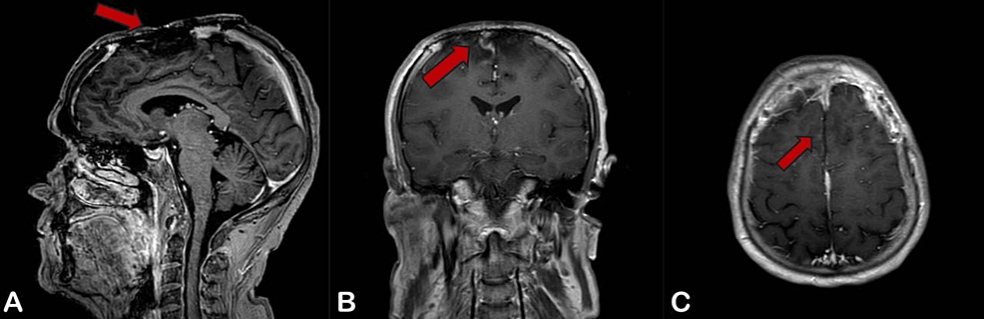
(A) Sagittal, (B) Coronal, and (C) Axial T2-Weighted MRI With Contrast, Showing Post-frontoparietal Cranioplasty for Meningioma Resection With Bilateral Frontal Encephalomalacia, Gliosis, and Small Left Parafalcine Residual Lesions.

Postoperative evaluation was done, and the patient was doing fine until a week later when a new swelling was noted. It showed a serum collection in the cavity, which was resolved by needle aspiration. A follow-up MRI with contrast three months later showed no further re-growth (Figure 5).

Continued surveillance with biannual MRI was recommended.

## Discussion

Meningiomas are benign tumors affecting almost 1% of the general population [4]. They develop from arachnoid cap cells, typically forming the arachnoid villi and the outer layer of the arachnoid mater. The exact process of unregulated arachnoid cap cell multiplication remains unknown. Genetic tests have demonstrated that a suppression mutation in neurofibromatosis type-2 (NF-2), an important tumor suppressor gene, is associated with up to 40% of instances of childhood meningioma [5]. Recent research indicates that insulin-like growth factor II (IGF-II) and epidermal growth factor receptor upregulation are vital in meningioma development. However, the genetic and molecular pathways are still being investigated [6].

The clinical manifestation of meningioma varies according to the tumor's size and location. Small meningiomas are usually asymptomatic. However, the mass effect caused by large meningiomas may lead to various neurological symptoms, most commonly headache and visual problems, which affect around 45% and 30% of patients with large meningiomas, respectively [7]. Meningiomas are most frequent in elderly persons, with the prevalence rising dramatically beyond the age of 65. They commonly affect females, with a female-to-male ratio of approximately 3:1 in young and middle-aged individuals. This gender disparity is significantly lower but still noticeable in the elderly. The literature reports a recent rise in the overall incidence of meningioma, which could reflect modifications in tumor identification and reporting [8].

A provisional diagnosis of meningioma is frequently made via radiologic identification. CT scan is usually the first investigation performed in case of a suspected cerebral mass lesion. Non-contrast CT shows meningiomas as solitary, well-defined isodense or slightly hyperdense masses. Tumors are extra-axial, with an extensive dural base. Large tumors can cause inward displacement of the cortical grey matter. Rarely is an infiltrative growth pattern across the dura seen, known as "meningioma en plaque." Edema is frequently present, appearing as a low-density area around the tumor. Arterial constriction can occur when the tumor engulfs the surrounding arteries. Calcifications can be found in more than 20% of meningiomas and may be inversely associated with tumor growth capacity; thus, robust calcification is likely to have a favorable impact [9,10]. In our case, a non-enhanced CT scan showed an anterior bilateral frontal extra-axial slightly hyperdense lesion with calcification.

MRI is the modality of choice for diagnosing and characterizing meningiomas. Tumors are well defined and can appear isointense or, less typically, hypointense on T1-weighted images. Meningiomas are generally isointense or hyperintense on T2-weighted sequences, but they can become hypointense compared to grey matter on rare occasions. Edema appears hypointense on T1-weighted sequences but hyperintense on T2-weighted sequences. Surprisingly, several studies show that the presence of intraaxial edema indicates a higher possibility of tumor regrowth [11]. However, meningioma's radiologic findings may be similar to other benign and malignant brain tumors [12,13]. As a result, a good history, medical examination, radiological findings, and biopsy results are crucial in diagnosing this common tumor [4].

The use of digital subtraction angiography (DSA) for the pre-operative evaluation of collateral circulation reveals features such as non-visualization of a segment of the superior sagittal sinus, non-reach of cortical veins, delayed vein emptying, and reversal of blood flow in collateral veins connecting the sinus with other venous outflow channels. The collateral venous circulation includes anastomoses between cortical veins and deep ventricular and cisternal veins, end-to-end anastomoses of superficial cortical veins, anastomoses with meningeal veins, and anastomoses with scalp veins [14-16]. Yin et al. aimed to study the venous compensatory patterns in 45 patients with meningioma invading the SSS. They concluded that pre-operative parafalcine collateral veins and falcine sinus assessment by MRV can help prevent venous iatrogenic injury during the operation, especially with complete posterior SSS occlusion [3].

Surgery for the management of parasagittal and falcine meningiomas has been associated with an increased risk of cerebral hemodynamic complications. Meningiomas in this region tend to invade the superior sagittal sinus and encase or attach to the large veins, making it difficult to safely and entirely remove the tumor. Several authors consider meningioma invasion of the superior sagittal sinus to be a contraindication to complete tumor excision because of the danger of harm to the cerebral venous circulation system during the attempt of radical resection [17,18]. As a result, in some studies, this group had the highest recurrence rate of all meningiomas [19,20]. Conversely, attempts at radical resection of the tumor to reduce the risk of recurrence have increased the risk of damage to the cerebral venous system, leading to several complications that can cause death in patients [21]. This necessitates the development of approaches for the surgical intervention of parasagittal and falcine meningiomas invading the SSS to eliminate clinical signs, control tumor growth, and prevent neurological deterioration postoperatively. 

The complications following meningioma surgery are often due to damage to the venous circulation within the superior sagittal sinus and its inflows. When the tumor is removed, the bridging veins entering the superior sagittal sinus may be affected. Losing a bridging vein enhances thrombosis within the damaged vessels by creating focal venous engorgement and decreased vascular flow, promoting venous stasis. The involvement of the large draining veins in thrombosis results in brain congestion and venous infarction [22,23]. Patients with intracranial meningiomas are also more likely to develop venous thrombosis due to the tumor's tendency to secrete tissue thromboplastin [24].

The therapeutic technique involves radical tumor resection and reconstruction of the SSS and its inflows. According to Sindou [17], this approach results in a recurrence rate of only 4%, a 3% mortality rate, and 8% of cases leading to neurological impairment, with higher-grade meningiomas accounting for half of the patients. An alternative technique is to perform partial resection and wait for the residual tumor to occlude the sinuses and promote collateral venous outflow. However, this strategy may be misleading because it cannot ensure control of tumor growth, which can continue down the sinus while maintaining its patency. Full closure of the SSS does not ensure tumor resection safety. Collateral circulation failure in meningiomas that infiltrate major venous sinuses can be challenging to prevent since it occurs in the dura mater at the surgical sites. Sindou's study of 100 patients found that all deaths and five of eight severe neurological consequences occurred following tumor removal without venous system reconstruction, resulting in brain swelling and venous infarction symptoms. Aggressive treatment of a tumor in the SSS is linked to a high risk of neurological complications, mainly when the sinus shows patency and is ligated and resected with the tumor, despite its use only for meningiomas in the anterior one-third part [1]. A study by Nowak et al. involved 37 patients who underwent surgery for meningiomas invading the SSS [1]. Thirteen patients underwent resection of the SSS with the tumor, 14 patients underwent tumor resection with SSS reconstruction, and in the last 10 patients, incomplete tumor resection was performed. The postoperative follow-up ranged from 24 months to 19 years. They noted that the safest postoperative technique was leaving the tumor in the SSS but recommended early use of stereotactic radiosurgery due to the high risk of recurrence. In contrast, aggressive surgical treatment of meningiomas that have invaded the SSS is associated with significant surgical risk but decreases the risk of recurrence [1].

Metastatic meningioma is uncommon, with right-to-left subfalcine herniation occurring in only about 0.2% of all meningiomas and in 40%-45% of aggressive subtypes of WHO grade III tumors [25]. In contrast, Beutler et al. described a case of a 78-year-old man with a WHO grade I meningioma who underwent surgery for tumor removal. Regular follow-up MRIs five years postoperatively showed recurrence of the meningioma, and two years later, he developed metastasis to the liver.

## Conclusions

Meningioma is the most frequently identified primary tumor of the brain, and the majority of these tumors are classified as benign. Meningiomas are often diagnosed presumptively with a CT scan and MRI, but a biopsy provides a definitive diagnosis. Surgical intervention for parasagittal and falcine meningiomas aims to inhibit tumor progression while preserving neurological function. There are several surgical approaches to treat these tumors, with the possibility of utilizing stereotactic radiosurgery.
